# Identification of Prothrombin Belgrade Variant in a Mexican–American Family with Recurrent Deep Vein Thrombosis

**DOI:** 10.1055/a-2766-5989

**Published:** 2025-12-22

**Authors:** Émile Moura Coelho da Silva, Natalie Montanez, Miguel Escobar

**Affiliations:** 1 Gulf States Hemophilia and Thrombophilia Center, McGovern Medical School, The University of Texas Health Science Center at Houston, Houston, Texas, United States; 2 Department of Neurology, McGovern Medical School, The University of Texas Health Science Center at Houston, Houston, Texas, United States

**Keywords:** F2 gene variant, prothrombin, thrombophilia, venous thromboembolism

## Abstract

A rare prothrombin variant (c.1787G > A, p.Arg596Gln), also known as the prothrombin Belgrade variant, has been associated with an increased predisposition to thrombosis through resistance to antithrombin. This variant has been previously reported in individuals from Serbia, Japan, China, and India. In this case report, we described the first reported case of heterozygosity for the prothrombin Belgrade variant in a Mexican–American family. Affected individuals had negative results on standard hypercoagulable studies; however, they exhibited a history of early-onset and recurrent venous thromboembolism (VTE). Although rare, the prothrombin Belgrade variant—and other prothrombin variants associated with antithrombin resistance—may be underrecognized in patients with recurrent thrombotic events, particularly among individuals from ethnic backgrounds not previously associated with this variant. These findings support the consideration of comprehensive genetic thrombophilia testing, including full sequencing of the prothrombin gene, in patients with negative standard hypercoagulable studies but a strong personal and/or family history of VTE.

## Introduction

Venous thromboembolism (VTE) affects approximately 1 in 1,000 individuals annually. 1 While VTE is multifactorial in origin, a hereditary cause should be suspected in patients with early-onset VTE (typically before age 40 or 50), a strong family history, recurrent episodes, or thrombosis in unusual locations, such as the central nervous system. 2 Standard hereditary thrombophilia evaluation includes testing for protein S, protein C, antithrombin deficiency, and the factor V Leiden and prothrombin G20210A variants. 3 However, additional genetic variants associated with thrombosis risk have been identified, including several variants in the prothrombin ( *F2* ) gene that confer antithrombin resistance. 4 5 6 One such variant is the prothrombin Belgrade (c.1787G > A, p.Arg596Gln), previously reported in individuals from Serbia, Japan, China, and India. 4 7 8 9 We report the first known case of this variant in a Mexican–American family with a history of recurrent VTE.

This study was conducted in accordance with the principles of the Declaration of Helsinki. As the work consists of case reports and does not constitute systematic research on human subjects, it was exempt from Institutional Review Board review, and individual informed consent was formally waived. Nevertheless, informed consent was obtained from two of the three participants; the third participant could not be reached despite multiple attempts.

## Case Summaries

### Case 1

A 35-year-old Mexican–American female with an initial thrombotic event at age 22: An extensive, unprovoked left lower extremity deep vein thrombosis (DVT) involving the left common femoral, superficial femoral, deep femoral, popliteal, posterior tibial, and peroneal veins. Initial hypercoagulable studies—including functional levels of fibrinogen, antithrombin, protein C and S, as well as testing for factor V Leiden, prothrombin G20210A variant, and antiphospholipid antibodies—were negative. She was initially treated with enoxaparin and later transitioned to long-term warfarin therapy.

Warfarin was discontinued during pregnancy, and the patient was managed with intermediate-dose enoxaparin (40 mg subcutaneously every 12 hours). At age 29, while still on enoxaparin antepartum, she developed superficial thrombophlebitis of the right upper extremity following peripheral catheter placement. In the postpartum period, after stopping anticoagulation prophylaxis, she experienced a right lower extremity distal DVT.

At age 31, off anticoagulation, she developed a recurrent non-occlusive thrombus in the left common femoral vein. Imaging revealed left iliac compression syndrome, necessitating thrombectomy, angioplasty, and venous stent placement. Six days later, she developed a recurrent thrombus in the left superficial femoral vein at the mid-thigh level, requiring repeat angioplasty and catheter-directed thrombolysis.

**Fig. 1:**
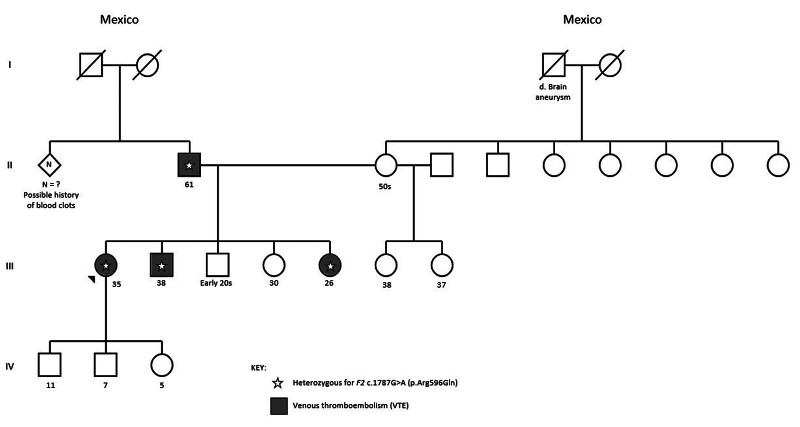
Family pedigree. Circles represent females and squares represent males. Solid (gray) symbols indicate family members affected with venous thromboembolism, open (white) symbols indicate unaffected members, and slashed symbols indicate deceased individuals. Stars denote heterozygosity for the prothrombin Belgrade variant ( *F2* c.1787G > A, p.Arg596Gln). Individuals II-2, III-1, III-2, and III-5 carry this variant.

Her family history is notable for recurrent DVT in her father, brother, and sister ( Fig. 1 ). In December 2022, comprehensive thrombophilia genetic testing—including full gene sequencing and deletion/duplication analysis of 11 genes ( *ADAMTS13* , *F2* , *F5* , *F9* , *FGB* , *FGG* , *MPL* , *PROC* , *PROS1* , *SERPINC1* , *THBD* )—identified a heterozygous pathogenic variant in the *F2* gene (NM_000506.3): Prothrombin Belgrade (c.1787G > A, p.Arg596Gln).

### Case 2

A 26-year-old Mexican–American female, the full sister of the patient described in Case 1, experienced her first thrombotic event at age 13. She presented with an extensive, unprovoked left lower extremity DVT, extending from the left iliac vein to the popliteal vein. Vascular imaging confirmed left iliac vein compression syndrome requiring thrombectomy, angioplasty, and venous stent placement.

Initial hypercoagulable studies—including functional assays for fibrinogen, antithrombin, protein C and S, and screening for factor V Leiden, prothrombin G20210A, and antiphospholipid antibodies—were negative. At age 21, while on low-dose antiplatelet prophylaxis, she developed a recurrent left proximal lower extremity DVT. She experienced further recurrent left lower extremity DVT at ages 22, 23, and 25, each occurring during periods of non-adherence or interruption in anticoagulation therapy.

In March 2023, after her sister was identified as heterozygous for the prothrombin Belgrade variant, the patient underwent targeted familial variant testing. She was found to be heterozygous for the same pathogenic variant in the *F2* gene (c.1787G > A, p.Arg596Gln).

### Case 3

A 38-year-old Mexican-American male, the full brother of the patients described in Cases 1 and 2, experienced his first thrombotic event at age 24. He presented with an extensive, unprovoked left lower extremity DVT, extending from the iliac vein to the popliteal vein. Vascular imaging ruled out iliac vein compression syndrome. He has remained on long-term anticoagulation and has persistent, chronic non-occlusive thrombi involving the left iliac, common femoral, superficial femoral, and popliteal veins. His clinical course has also been complicated by venous ulceration on the affected extremity.

In March 2023, following the identification of the prothrombin Belgrade variant in his sisters, he underwent targeted familial variant testing and was found to be heterozygous for the same *F2* gene variant (c.1787G > A, p.Arg596Gln).

The siblings' father has a reported history of recurrent lower extremity DVT. He also underwent targeted genetic testing and was found to be heterozygous for the prothrombin Belgrade (c.1787G > A, p.Arg596Gln) variant.

## Discussion

Prothrombin ( *F2* ) is the precursor of thrombin, a serine protease that plays a central role in hemostasis. 10 The interaction between thrombin and antithrombin is critical to maintaining anticoagulant balance. Residue Arg596 of prothrombin lies within a key antithrombin-binding site required for thrombin inactivation. 4 6 Variants affecting Arg596 have been shown to disrupt this interaction, impairing thrombin inhibition by antithrombin and resulting in antithrombin resistance and a prothrombotic state. 4 5 6 Reported variants at this position include prothrombin Yukuhashi (p.Arg596Leu), Padua 2 (p.Arg596Trp), and Belgrade (p.Arg596Gln). The latter, also referred to as prothrombin Amrita, has been previously identified in individuals from Serbia, China, India, and Japan. 4 7 8 9 11 12

The prothrombin Belgrade variant is associated with early-onset VTE, with a reported mean age of first thrombotic event at 28.4 years (range 15–67 years). 11 Most affected individuals also report recurrent VTE and a strong family history of thrombotic disease. Heterozygous carriers of the Arg596 variants manifest a pronounced prothrombotic phenotype, consistent with the autosomal dominant inheritance of this condition. This strong effect is likely due to the critical enzymatic role of the Arg596 residue in thrombin's interaction with antithrombin. 4 5 6 10 13 In heterozygotes, the presence of one mutant allele produces thrombin molecules with impaired antithrombin binding, effectively reducing the overall inhibitory control over thrombin activity. 4 5 6 13 Because thrombin functions in a catalytic, amplification-driven cascade, even a partial reduction in its regulation can disproportionately increase coagulation potential, leading to thrombophilia. 10

While the prothrombin G20210A variant is found in approximately 2% of the U.S. population, prothrombin variants associated with antithrombin resistance are estimated to affect less than 1% of individuals. 13 14 However, the true prevalence of antithrombin resistance may be underestimated due to limitations in standard thrombophilia testing protocols. Conventional hereditary thrombophilia workup typically includes assays for protein C, protein S, and antithrombin, and factor V Leiden and prothrombin G20210A variants. 3 Full sequencing of the *F2* gene is not routinely performed, and as a result, rarer pathogenic variants such as prothrombin Belgrade may go undetected.

Here, we describe three Mexican–American siblings with extensive personal and family history of VTE who were found to be heterozygous for the prothrombin Belgrade variant following initial negative standard hypercoagulable evaluations. This represents the first reported family of Mexican–American descent with this variant. These cases highlight the need to consider pursuing comprehensive genetic thrombophilia testing, including full *F2* gene sequencing, in patients with unexplained, recurrent, or early-onset VTE and a positive family history—especially when conventional thrombophilia studies are unrevealing. Identifying a molecular diagnosis can inform long-term management, guide cascade testing in at-risk relatives, and support risk-reduction strategies, ultimately improving patient and family outcomes.
